# Ketamine Metabolites Enantioselectively Decrease Intracellular D-Serine Concentrations in PC-12 Cells

**DOI:** 10.1371/journal.pone.0149499

**Published:** 2016-04-20

**Authors:** Nagendra S. Singh, Ewelina Rutkowska, Anita Plazinska, Mohammed Khadeer, Ruin Moaddel, Krzysztof Jozwiak, Michel Bernier, Irving W. Wainer

**Affiliations:** 1 Laboratory of Clinical Investigation, National Institute on Aging, National Institutes of Health, Baltimore, MD, 21224, United States of America; 2 Department of Biopharmacy, Medical University of Lublin, Lublin, Poland; 3 Translational Gerontology Branch, National Institute on Aging, National Institutes of Health, Baltimore, MD, 21224, United States of America; Aston University, Birmingham, UNITED KINGDOM

## Abstract

D-Serine is an endogenous NMDA receptor co-agonist that activates synaptic NMDA receptors modulating neuronal networks in the cerebral cortex and plays a key role in long-term potentiation of synaptic transmission. D-serine is associated with NMDA receptor neurotoxicity and neurodegeneration and elevated D-serine concentrations have been associated with Alzheimer’s and Parkinsons’ diseases and amyotrophic lateral sclerosis. Previous studies have demonstrated that the ketamine metabolites (*rac*)-dehydronorketamine and (2*S*,6*S*)-hydroxynorketamine decrease intracellular D-serine concentrations in a concentration dependent manner in PC-12 cells. In the current study, PC-12 cells were incubated with a series of ketamine metabolites and the IC_50_ values associated with attenuated intracellular D-serine concentrations were determined. The results demonstrate that structural and stereochemical features of the studied compounds contribute to the magnitude of the inhibitory effect with (2*S*,6*S*)-hydroxynorketamine and (2*R*,6*R*)-hydroxynorketamine displaying the most potent inhibition with IC_50_ values of 0.18 ± 0.04 nM and 0.68 ± 0.09 nM. The data was utilized to construct a preliminary 3D-QSAR/pharmacophore model for use in the design of new and more efficient modulators of D-serine.

## Introduction

(*rac*)-Ketamine (Ket) is a chiral phencyclidine derivative that produces rapid and short-lived anesthesia via inhibition of the N-methyl-D-aspartate (NMDA) receptor [1,2]. (*rac*)-Ket is rapidly, extensively and stereoselectively transformed into an array of N-demethylated and hydroxylated metabolites including norketamine (norKet), dehydronorketamine (DHNK) and diastereomeric hydroxynorketamines (HNK) [3,4]. Initial studies of (*rac*)-Ket induced anesthesia demonstrated that the effect was produced by the parent compound and (*R*,*S*)-norKet and that the (2S,6S;2R,6R)-HNK metabolite was inactive in this test [5]. However, recent studies have demonstrated that while the HNK and DHNK metabolites may not significantly contribute to the anesthetic effects of Ket, they are associated with the antidepressant effects in patients suffering from treatment resistant major depressive disorder (MDD) and bipolar depression produced by subanesthetic dosing of (R,S)-Ket [6]. In addition, administration of (2*S*,6*S*)-HNK stimulated phosphorylation of the mammalian target of rapamycin (mTOR) and its downstream targets in Wistar rat pre-frontal cortex tissue [7]. This effect was also observed after the administration of (R,S)-Ket and was associated with (R,S)-Ket’s antidepressant activity in the Wistar rat [8,9].

We have demonstrated that racemic (2*S*,6*S*;2*R*,6*R*)-HNK, (2*S*,6*R*;2*R*,6*S*)-HNK and (*rac*)-DHNK have a low affinity for the NMDA receptor [10], and, as a result, it is unlikely that the direct inhibition of the NMDA receptor is the source of the observed activation of the mTOR pathway. A potential pharmacological mechanism explaining mTOR activation is an “indirect” inhibition of NMDA receptor produced by a reduction in the concentration of D-serine. D-Serine, an endogenous NMDA receptor co-agonist, plays a critical role in long-term potentiation and NMDA-induced neurotoxicity and a decrease in D-serine concentration has been associated with reduced NMDA receptor activity [11–14]. We have previously demonstrated that incubation of PC-12 pheochromocytoma and 1321N1 astrocytoma cells with (*rac*)-DHNK and (2*S*,6*S*)-HNK reduces intracellular D-serine concentrations [7,15]. Paradoxically, these compounds also stimulate mTOR signaling resulting in increased expression of serine racemase (SR), the enzyme that mediates racemization of L-serine to D-serine. The effect of (2*S*,6*S*)-HNK and (R,S)-Ket on mTOR signaling in PC-12 and 1321N1 cells was similar to the effect observed was in rat pre-frontal cortex tissues [7].

The *in vitro* effects of the HNK and DHNK metabolites are consistent with results obtained in MDD patients. In these patients, antidepressant response to treatment with (R,S)-Ket was associated with pre-dose plasma D-serine concentrations, as basal D-serine levels are significantly lower in MDD patients that respond to treatment relative to non-responders [16]. The administration of (R,S)-Ket resulted in a ~20–25% decrease in D-serine plasma levels immediately following its 40-min infusion followed by a recovery to pre-dose levels at 120 min and then a slow decreased over the next 24h [16]. The rapid fall in plasma D-serine levels is clinically relevant as it is associated with increased dissociative side effects reflected as increased scores on the Clinician-Administered Dissociative States Scale (CADDS) which peak at 40 min after the initiation of the (*R*,*S*)-Ket infusion and return to baseline at 80 min [16,17]. We have recently demonstrated that the rapid decrease in plasma D-serine concentrations was produced by (*S*)-Ket inhibition of the alanine-serine-cysteine transporter (ASCT2), which mediates D-serine cellular export, while the latter, slower decease in D-serine plasma concentrations has been attributed to the activities of (*R*,*S*)-Ket metabolites [17].

**Fig 1:**
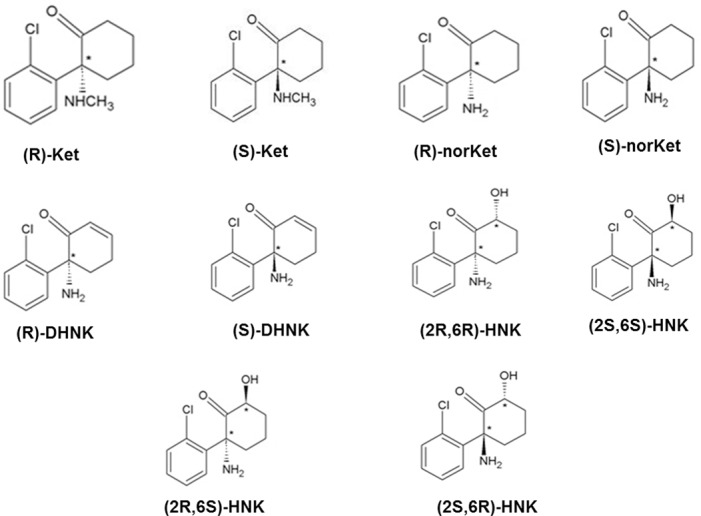
The structures of the compounds used in this study.

Since D-Serine concentrations have been correlated with a number of CNS diseases such as amyotrophic lateral sclerosis (ALS), Alzheimer’s and schizophrenia, the development of drugs that can modulate D-serine expression and distribution is an area of pharmacological and clinical interest [18,19]. The data from our previous studies of the effect of (R,S)-DHNK and (2*S*,6*S*)-HNK on intracellular D-serine concentrations suggest that these compounds might be a starting point for a D-serine-targeted drug discovery program. We now report the expansion of our initial observations through the determination of the effect of the individual enantiomers of (*R*)- and (*S*)-norKet, (*R*)- and (*S*)-DHNK, (2*S*,6*S*)- and (2*R*,6*R*)-HNK and (2*S*,6*R*)- and (2*R*,6*S*)-HNK, Fig 1, on intracellular D-serine concentration and SR expression in PC-12 cells. The limited data set demonstrated that structural, steric and stereochemical factors contributed to the modulation of intracellular D-serine concentration and SR expression. The data was utilized to construct a preliminary 3D-QSAR/pharmacophore model for use in the design of new and more efficient modulators of D-serine.

## Materials and Methods

### Ketamine (Ket) Metabolites

The Ket metabolites used in this study were (*R)*- and *(S)*-norKet, (*R)*- and *(S)*-DHNK, (*2R*,*6R)*- and (*2S*,*6S)*-HNK, and (*2R*,*6S)*- and (*2S*,*6R)*-HNK. These metabolites were prepared as previously described [20].

### Maintenance and Treatment of Cell Lines

The PC-12 cell line, which is derived from rat adrenal medulla, was obtained from American Type Culture Collection (Manassas, VA, USA). The PC-12 cells were maintained in RPMI-1640 (Quality Biological, Gaithersburg, MD, USA) supplemented with 4-(2-hydroxyethyl)-1-piperazineethanesulfonic acid (HEPES) buffer [1 mM, pH 7.4] (Mediatech, Inc., Manassas, VA, USA), 10% heat-inactivated horse serum (Biosource, Rockville, MD, USA), 5% fetal bovine serum (FBS), 1% sodium pyruvate, 5% L-glutamine and 1% penicillin/streptomycin all purchased from Quality Biological.

### Incubation of PC-12 Cells with Ket Metabolites

Cells were seeded on 100 x 20 mm tissue culture plates and maintained at 37°C under humidified 5% CO_2_ in air until they reached >70% confluence. Serial dilutions of the test compounds were prepared in the media using stock solutions of the compounds (10 mM in ethanol). The original media was replaced with media containing the test compounds and the plates were incubated for an additional 36 h, unless otherwise indicated. The medium was removed, and the cells collected for analysis. In the first series of experiments, (*R*)-norKet and (*S*)-norKet (0–1μM); (*R*)-DHNK and (*S*)-DHNK (0–0.45μM); *(2R*,*6R)*-HNK, *(2S*,*6S)*-HNK, *(2R*,*6S)*-HNK, and *(2S*,*6R)*-HNK (0–0.1μM) were tested. The cells were assessed for intracellular and extracellular D-serine levels, and expression of monomeric and dimeric forms of serine racemase (SR). The intracellular D-serine levels were determined in triplicate dishes while the determination of SR protein expression was carried out on one set of dishes. Additionally, extracellular D-serine levels were determined in cells treated with *(2S*,*6S)*-HNK. All analyses were repeated in three independent cell cultures (n = 3).

### Determination of Intracellular and Extracellular D-Ser Concentrations

Intracellular D-serine concentrations were measured using a previously described and validated capillary electrophoresis-laser induced fluorescence (CE-LIF) method using a P/ACE MDQ system equipped with a laser-induced fluorescence detector (Beckman Instruments, Fullerton, CA, USA) [21]. The extracellular D-serine levels were determined using a previously reported assay employing liquid chromatography with mass spectrometric detection [17].

### Measurement of Monomeric-SR (m-SR) and Dimeric-SR (d-SR) Expression by Western Blotting

The expression of m-SR and d-SR in PC-12 cells was determined using a previously described procedure [15]. The primary antibody for d-SR was obtained from Santa Cruz Biotechnology (Dallas, TX, USA), and the antibody that recognizes both m-SR and d-SR was purchased from Abcam, Inc. (Cambridge, MA). The primary antibody for β-actin was from Abcam. The antibodies were used at a dilution recommended by the manufacturer. Immunoreactive bands were detected using the ECL Plus Western Blotting Detection System (GE Healthcare, Piscataway, NJ, USA) and quantification was accomplished by volume densitometry using ImageJ software (National Institutes of Health, Bethesda, MD) and normalization to β-actin.

### Comparative Molecular Field Analysis (CoMFA)

The CoMFA model was generated using methodology implemented in Sybyl-X 2.1.1 (Certara, L.P., Princeton, NJ, USA). The molecular models of structures were prepared in HyperChem v. 6.03 (HyperCube Inc., Gainesville, FL) using Model Build procedure to ensure the same conformation of the common scaffold. The models were extracted to Sybyl and the Gasteiger-Huckel atomic charges were calculated. The models were aligned using 2-chlorobenzyl moiety as a common substructure. Two types of molecular fields (steric and electrostatic) were sampled on the grid lattice surrounding each structure. In the procedure default settings were used. The pIC_50_ values presenting effects on the intracellular D-serine levels in PC-12 cells of ketamine metabolites were subjected to 3D-QSAR modeling

### Statistical Analysis

Prism 4 (GraphPad Software, Inc., La Jolla, CA, USA) running on a personal computer was used to perform all statistical data analysis, including IC_50_ value calculations. The effect of test compounds on intracellular D-serine concentration is reported as ‘average percent change ± standard deviation’ compared to control values. Differences between two groups were analyzed using Student’s t-test (unpaired, two-tailed). A *P* value ≤ 0.05 was considered significant.

## Results

### Ket Metabolites Reduce Intracellular D-Serine Concentrations

The molecular structures of the compounds used in this study are presented in Fig 1. All of the tested compounds, (*R*)-norKet, (*S*)-norKet, (*R*)-DHNK, (*S*)-DHNK, (*2S*,*6S*)-HNK, (*2R*,*6R*)-HNK, (*2S*,*6R*)-HNK and (*2R*,*6S*)-HNK significantly reduced intracellular D-serine concentration. The maximum effects, determined as percent decrease from control, were produced by (*2S*,*6R*)-HNK and (*2R*,*6S*)-HNK with reductions of 51% and 57%, respectively with the remaining decreases in the range of 29% ((*S*)-norKet) to 39% ((*2R*,*6R*)-HNK) (Table 1). The reductions in intracellular D-serine levels were concentration-dependent and the potency of the compounds, expressed as IC_50_ value, ranged from ~100nM to 0.18nM (Table 1).

**Table 1:** The concentration-dependent decrease of intracellular D-Ser concentrations produced by incubation of PC-12 cells with the enantiomers of ketamine (Ket) and its major metabolites expressed as IC_50_ values and percent (%) maximum decrease from vehicle-treated PC-12 cells. The effect of the configuration at the chiral centers on the magnitude of the IC_50_ value represented as the enantioselectivity factor α is derived by IC_50_(2R isomer)/IC_50_(2S isomer). The values for (*R*)-Ket were obtained from [17] and ‘NA’ indicates that (*S*)-Ket increases intracellular D-Ser concentrations [17]. Results are expressed as means ± SD, n = 3 independent experiments.

Compound	IC_50_ (nM)	Decrease	Compound	IC_50_ (nM)	Decrease	(α)
(*R*)-Ket	940 ± 160	-33 ± 8%	(S)-Ket	NA	NA	NA
(*R*)-norKet	91.0 ± 5.9	-39 ± 2%	(S)-norKet	62.4 ± 1.0	-29 ± 1%	1.5
(*R*)-DHNK	102 ± 11	-32 ± 9%	(S)-DHNK	50.9 ± 6.2	-30 ± 2%	2.0
(*2R*,*6R*)-HNK	0.68±0.09	-35 ± 3%	(2S,6S)-HNK	0.18 ± 0.04	-39 ± 5%	3.8
(*2R*,*6S*)-HNK	2.34±0.32	-57 ± 5%	(2S,6R)-HNK	1.11 ± 0.24	-51 ± 4%	2.1

### The Attenuation of Intracellular D-Serine Concentration is Stereospecific

The stereochemical configuration at the C2 carbon on the cyclohexanone ring affected the relative IC_50_ values associated with the decrease in intracellular D-serine concentration (Table 1). The metabolites with an *S*- configuration at the C2 carbon were more potent than the corresponding *R*-isomers, and the enantioselectivity determined as the ratio of the IC_50_ of the *R*-isomer to that of the *S*-isomer ranged from 1.5 ((*R*/*S*)-norKet) to 3.8 ((2*R*,6*R*/*2S*,*6S*)-HNK) (Table 1).

The addition of the hydroxyl moiety at the C6 position in the cyclohexanone ring of norKet produces a second chiral center and a significant increase in the inhibitory activity as measured by decreased IC_50_ values (Table 1). This effect was produced regardless of the configuration at the C2 or C6 position, but there was an important relationship between activity and the stereochemistry at two chiral centers. In the HNK molecules, a *cis* stereochemical relationship between the C2 and C6 chiral centers on the cyclohexanone ring, i.e. (*2S*,*6S*)-HNK and (2*R*,6*R*)-HNK, produced a more potent inhibition when compared to the corresponding HNK metabolites with a *trans* relationship between the two chiral centers. The diastereoselective effect on intracellular D-serine concentration (α_D_) ranged from 1.6 to 13, calculated as IC_50_(*trans*-isomer)/IC_50_(*cis*-isomer) (Table 2).

**Table 2:** The stereochemical configurations at the C2 and C6 carbons of hydroxynorketamine (HNK) affect the concentration-dependent decrease in intracellular D-Ser concentration in PC-12 cells. The relative pharmacological activity (α_D_) is derived by IC_50_(*trans*-isomer)/IC_50_(*cis*-isomer). The IC_50_ values are expressed as means ± SD, n = 3 independent experiments.

Compound	IC_50_ (nM)	Compound	IC_50_ (nM)	Stereoselectivity (α_Δ_)
(*2R*,*6R*)-HNK	0.68 ± 0.09	(2R,6S)-HNK	2.34 ± 0.32	3.4
(*2R*,*6R*)-HNK	0.68 ± 0.09	(2S,6R)-HNK	1.11 ± 0.24	1.6
(*2S*,*6S*)-HNK	0.18 ± 0.04	(2S,6R)-HNK	1.11 ± 0.24	6.2
(*2S*,*6S*)-HNK	0.18 ± 0.04	(2R,6S)-HNK	2.34 ± 0.32	13

### Ket Metabolites Increase SR Expression Is a Stereospecific Manner

**Fig 2:**
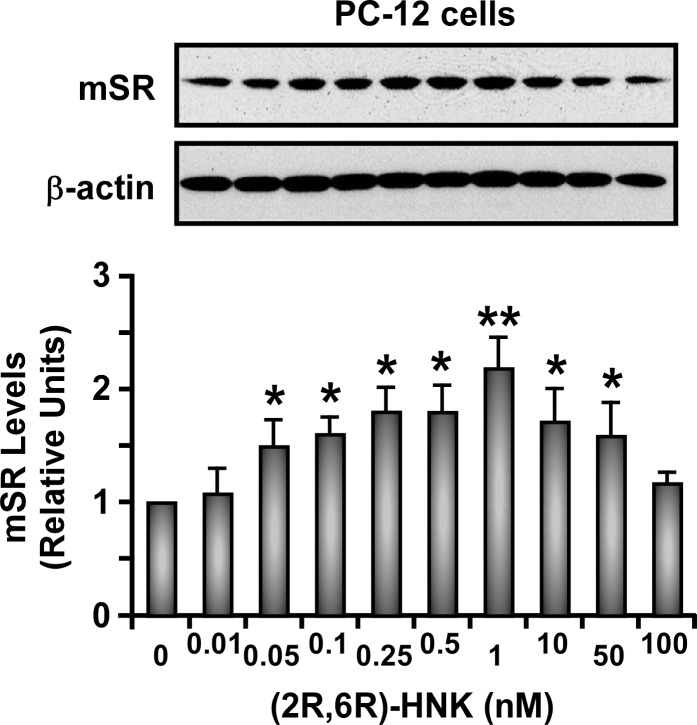
Effect of (2R,6R)-hydroxynorketamine (HNK) on the levels of serine racemase protein in PC-12 cells. Cells were treated with various concentrations of HNK for 36 h and then total cell lysates were prepared for Western blot analysis. Top panel: representative immunoblot of monomeric serine racemase (m-SR; 36-kilodalton band). The blot was reprobed for β-actin, which was used as loading control. Bottom panel: relative levels of m-SR after quantification and normalization with β-actin. Bars represent the means ± SD of three independent experiments. * *P< 0*.*05* and ** *P< 0*.*01* as compared with control cells.

We recently demonstrated that incubation of PC-12 cells with (*R*,*S*)-Ket, (*R*)-Ket, (*S*)-Ket, (*R*,*S*)-norKet, (*R*,*S*)-DHNK or (*2S*,*6S*)-HNK produced an ∼2-fold increase in the expression of the monomeric form of SR (m-SR) in a concentration-dependent inverted-U-shaped manner [7,15,17]. The effect was enantioselective with respect to (*S*)-Ket and (*R*)-Ket, as the minimum concentration required to elicit the maximum response with (*S*)-Ket was 200 nM while (*R*)-Ket required a concentration of 4,000 nM [17]. Here, similar inverted-U-shaped response curves were observed for all of the compounds examined in this study (Fig 2). The concentrations required to elicit maximal expression of m-SR ranged from 100 nM ((*R*)-norKet) to 0.25 nM ((2*S*,6*S*)-HNK) and were enantioselective as the compounds with an *S*- configuration at C2 were more potent than the corresponding enantiomer (Table 3).

**Table 3:** The minimum concentration (nM) of ketamine (Ket) and Ket metabolites required to elicit maximum increase in the expression of the monomeric form of SR (m-SR) in PC-12 cells. The effect of the configuration at the chiral centers at saturating concentration of each compound is represented as the enantioselectivity factor α derived from Concentration(2R isomer)/Concentration(2S isomer). The values for (R)-Ket and (S)-Ket were obtained from [17].

Compound	Concentration (nM)	Compound	Concentration (nM)	Enantioselectivity (α)
(*R*)-Ket	4,000	(S)-Ket	200	20
(*R*)-norKet	100	(S)-norKet	25	4
(*R*)-DHNK	75	(S)-DHNK	25	3
(*2R*,*6R*)-HNK	1	(2S,6S)-HNK	0.25	4
(*2R*,*6S*)-HNK	1	(2S,6R)-HNK	0.50	2

### (*2S*,*6S*)-HNK Does Not Inhibit ASCT2 Transport of D-Serine

We have previously shown that incubation of PC-12 cells with (*S*)-Ket increased the amount of intracellular D-serine by ∼59% while decreasing extracellular D-serine levels by ∼41% [17]. This effect was due to the enantioselective inhibition of ASCT2-mediated cellular export of D-serine. (*R*)-Ket had no effect on ASCT2 export of D-serine and incubation of PC-12 cells with (*R*)-Ket resulted in equivalent decreases in intracellular and extracellular D-serine concentrations [17]. In order to determine if inhibition of ASCT2 activity contributed to the observed results, PC-12 cells were incubated with varying concentrations of (*2S*,*6S*)-HNK, the most potent of the tested metabolites. A concentration-dependent reduction in extracellular D-serine levels was observed with a maximum decrease of 33.5 ± 2.5% and an IC_50_ value of 0.21 ± 0.08 nM (Table 4) as compared to the effect on intracellular D-serine concentration where the maximum decrease was 39 ± 5% and an IC_50_ value of 0.18 ± 0.04 nM (Table 1). The results indicate that (*2S*,*6S*)-HNK had no significant effect on ASCT2-mediated cellular export of D-serine. Since this stereoisomer is the most potent metabolite of (*S*)-Ket it is reasonable to assume that the other compounds examined in this study also do not inhibit ASCT2-mediated transport of D-serine.

**Table 4:** The concentration-dependent decrease of extracellular D-serine concentrations produced by incubation of PC-12 cells with the (2S,6S)-HNK and the associated IC_50_ value. The results are expressed as percent (%) maximum decrease from vehicle-treated PC-12 cells, means ± SD, n = 3 independent experiments.

	% Change in Extracellular D-Serine levels in PC-12 cells
**Conc. (nM)**	**(*2S*,*6S*)-HNK**
0	100.00
0.01	98.23 ± 0.30
0.05	91.40 ± 2.52
0.1	87.77 ± 1.99
0.25	81.86 ± 3.00
0.5	76.36 ± 3.25
1	73.40 ± 1.18
10	71.47 ± 0.79
50	67.89 ± 0.91
100	66.47 ± 1.52
**IC**_**50**_ **(nM)**	**0.21 ± 0.08**

### Relationship between Structure and Pharmacological Effect

**Fig 3:**
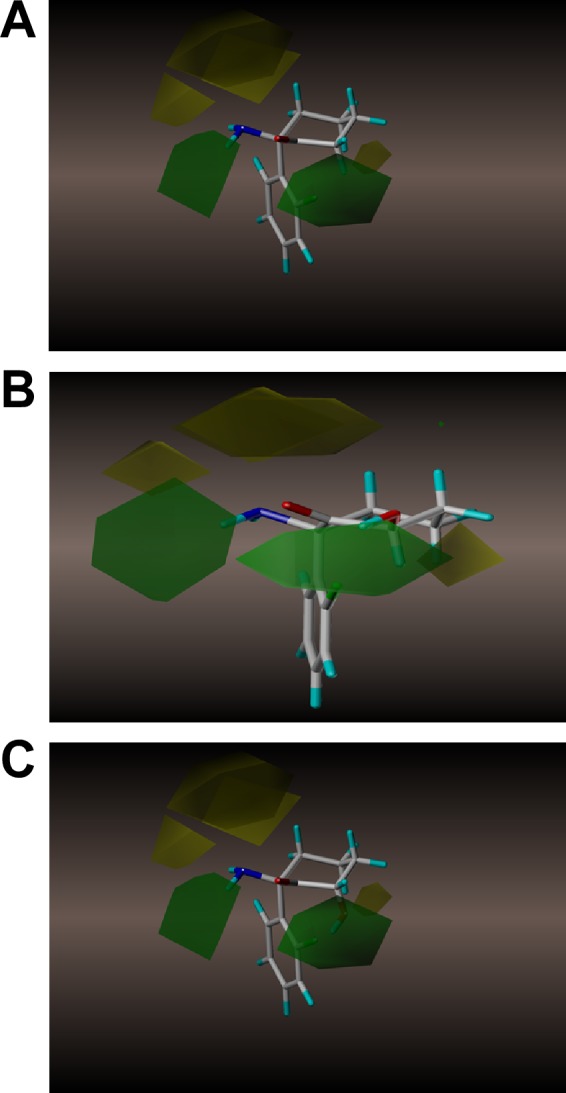
CoMFA models derived from the potency (determined as IC_50_ value) of ketamine metabolites on intracellular D-Ser concentrations. Nine compounds were considered in this study. The inclusion of (R)-norKet (**A**); (2R,6R)-HNK (**B**) and (2R,6S)-HNK (**C**) are depicted.

Although there were a limited number of compounds, a comparative molecular field analysis (CoMFA*)* was performed using the IC_50_ values and corresponding molecular structures. The resulting model identified several steric fields around the studied molecules that reached statistical significance in the analysis (Fig 3). The placement of (*R*)-norKet on the CoMFA model (Fig 3A) shows two fields, one sterically favorable (depicted in green) located on the left and another sterically unfavorable (yellow) on the right of the molecule. The model suggests that the N-methyl substituent on (*R*)-Ket would occupy a sterically unfavorable space, depicted as the top yellow region, resulting in a weak inhibition of D-serine production and that the removal of the N-methyl moiety relieves the steric interaction producing the stronger inhibitory effects observed with (*R*)-norKet and (*R*)-DHNK. The lack of a significant difference between the IC_50_ values of (*R*)-norKet and (*R*)-DHNK (Table 1) indicates that the effect on the conformation of cyclohexanone ring and electronic distribution at the carbonyl moiety produced by the introduction of the C5-C6 double bond had no impact on pharmacological activity. The inhibitory potencies of the norKet and DHNK molecules were enhanced by inversion of the stereochemistry at C2 as the IC_50_ values of the respective S-enantiomers, which strongly suggest that the cyclohexanone ring is in a favorable steric environment when the C2 carbon is in an S configuration.

The addition of a hydroxyl moiety at the C6 position of norKet dramatically increased the potency of the resulting HNK compounds with IC_50_ values that were from 40-fold to 300-fold lower than norKet (Table 1). This suggests that an additional stabilizing interaction, most probably hydrogen bonding, occurs between the HNK molecule and the target protein as depicted in the placement of (*2R*,*6R*)-HNK on the CoMFA model (Fig 3B). The observed difference in the IC_50_ values between the *cis* and *trans* isomers are consistent with the closer proximity to the sterically favorable spaces produced by the *cis* orientation as illustrated by a comparison of (*2R*,*6R*)-HNK and (*2R*,*6S*)-HNK in the CoMFA model (Fig 2B and 2C, respectively).

## Discussion

D-Serine and glycine are endogenous NMDA receptor co-agonists that act on different NMDA receptor populations [13,22]. Glycine has a demonstrated preference for extrasynaptic NMDA receptors associated with long term depression of synaptic signaling [13], while D-serine activates synaptic NMDA receptors modulating neuronal networks in the cerebral cortex [23] and plays a key role in long-term potentiation of synaptic transmission [12,13]. Recent studies have associated D-serine with NMDA receptor neurotoxicity and neurodegeneration [11,24] and elevated D-serine concentrations have been associated with Alzheimer’s and Parkinsons’ diseases and ALS [18,19]. In ALS, it has been suggested that increased levels of D-serine in the CNS is the primary cause of neuronal death associated with the disease [25]. In Alzheimer’s disease, elevated D-serine concentrations have been associated with increased expression of SR induced by amyloid β-peptide (Aβ_1–42_) [26] and secreted amyloid precursor protein [27].

The association of elevated D-serine with a number of CNS diseases and pathological states has resulted in drug development programs aimed at modulating the endogenous concentrations of this compound [18,19]. Since, the primary source of endogenous D-serine is SR-mediated racemization of L-serine these programs have targeted this enzyme using competitive and suicidal inhibitors [18,19]. SR is a pyridoxal-5’-phosphate-dependent enzyme whose activation is dependent upon the binding of divalent cations such as Mg^2+^ and Ca^2+^ to a metal binding site on the molecule [19] and intracellular Ca^2+^ concentrations affect the production of D-Ser [19,28,29]. For example, incubation of rat cortical astrocytes with the calcium ionophore A23187 increases D-Ser secretion [28] while the addition of a calcium chelator to the incubation media decreases D-Ser release from rat neuronal cultures [29]. The sensitivity of SR to changes in intracellular Ca^2+^ concentration suggests that the development of small molecule drugs designed attenuate intracellular Ca^2+^ concentration is a viable approach to the treatment of D-serine-related diseases. This approach is supported by our recent observation that the treatment of PC-12 cells with gabapentin and (*S*)-pregabalin produced significant decreases in intracellular D-Ser concentrations [30]. This effect was attributed to decreased intracellular Ca^2+^ flux resulting from the interaction of gabapentin and (*S*)-pregabalin with the α_2_-δ subunit of the voltage-gated Ca_v_α_2-_δ calcium channel [31].

We have recently demonstrated that incubation of PC-12 cells with (*rac*)-DHNK and (*2S*,*6S*)-HNK decreases the intracellular concentration of D-serine [7,15]. This effect was associated with the negative allosteric modulation of α_7_-nAChR activity, which results in lower intracellular Ca^2+^, which, in turn, reduces the magnitude of Ca^2+^–activated SR and consequently the intracellular D-Ser concentrations [7,10,15]. In the current study, we investigated the effect of the structure and stereochemistry of a series of Ket metabolites on the IC_50_ values associated with the decrease in intracellular D-serine in PC-12 cells. When compared to the IC_50_ value previously determined for (*R*)-Ket [17], the IC_50_ values of the N-demethylated metabolites, (*R*)-norKet and (*R*)-DHNK, were reduced by ~10-fold (Table 1). Hydroxylation of (*R*)-norKet at the C6 position on the cyclohexanone ring further reduced the IC_50_ values relative to (*R*)-Ket by more than 1400-fold for (*2R*,*6R*)-HNK and ~400-fold for (*2R*,*6S*)-HNK. A similar comparison for (*S*)-Ket was not possible as the incubation of PC-12 cells with (*S*)-Ket increased the intracellular pool of D-serine through enantioselective inhibition of ASCT2-mediated cellular export of D-serine [17]. However, the IC_50_ values of (*2S*,*6S*)-HNK and (*2S*,*6R*)-HNK were reduced by 344% and 56%, respectively, relative to (*S*)-norKet (Table 1).

The results of this study also indicate that the molecular structure of the HNK metabolites presents a template for the development of new and potent modifiers of endogenous concentrations of D-serine for use in the treatment of depression, Alzheimer’s disease, ALS and Parkinson’s disease. The initial CoMFA model derived from this data is a positive step in this direction. The potential clinical use of these compounds is supported by recent studies of the metabolism and disposition of (*2S*,*6S*)-HNK in the Wistar rat [32]. The results demonstrate that (*2S*,*6S*)-HNK is rapidly distributed with a volume of distribution (Vd) of 7352 ±736 ml.kg^-1^ and a half-life of drug elimination during the terminal phase (*t*_1/2_) of 8.0 ± 4.0 h. Significant concentrations of (2*S*,6*S*)-HNK are present in brain tissue samples 10 min after an intravenous administration and the compound has an oral bioavailability of 46.3%. The antidepressant activities of (*2S*,*6S*)-HNK and other Ket metabolites are under investigation in a number of mouse models and the data will be reported elsewhere. The data from the functional studies and the initial CoMFA model will be used to direct the synthesis of compounds in the next iteration in our program to develop highly effective and selective therapies.
